# Translating patient needs into medical device development: co-design of a photoprotection visor for Xeroderma Pigmentosum using qualitative interviews

**DOI:** 10.1186/s13023-023-03002-y

**Published:** 2024-02-13

**Authors:** Tanya Graham, Sangeeta Sooriah, Yan-Shing Chang, Shaikh Hashimdeen, Turgut Meydan, Patricia Grocott

**Affiliations:** 1 Florence Nightingale Faculty of Nursing, Midwifery and Palliative Care, Kings College London, James Clerk Maxwell Building, 57 Waterloo Road, London, SE1 8WA UK; 2 Healthwatch Camden, 85-87 Bayham Street, London, NW1 0AG UK; 3 University of Wales Trinity Saint David, Centre for Advanced Batch Manufacture (CBM), Waterfront, Innovation Quarter Campus, Heol Ynys, Kings Road, Swansea, SA1 8EW UK; 4 Cardiff School of Engineering, Cardiff University, Queen’s Buildings, The Parade, Cardiff, CF24 3AA UK

**Keywords:** Xeroderma Pigmentosum, Medical device development, Usability, Experienced-based codesign, Photoprotection, DNA damage, Dermatology, Skin cancer, Qualitative research

## Abstract

**Introduction:**

People with Xeroderma Pigmentosum (XP) have a heightened sensitivity to ultraviolet radiation (UVR) and are advised to wear photoprotective clothing including a visor covering the face and neck. Photoprotective visors are homemade and predominately worn by children with decreasing frequency as age increases. To improve upon the current design and efficacy we were tasked with developing a prototype visor to meet patients’ needs.

**Methods:**

Adopting a codesign methodology, patients’ experiences of wearing a visor and patient and carer views of emerging prototypes were explored during interviews. A thematic analysis was conducted in parallel with data collection and themes were interpreted into design cues; desirable attributes of a visor that would counteract the negative user experiences and meet the requirements described by patients and carers. The design cues guided the iterative development of prototypes by academic engineers.

**Results:**

Twenty-four interviews were conducted with patients and carers. Thematic analysis resulted in the following five themes: Being safe from UVR exposure; self-consciousness; temperature effects; acoustic difficulties; and material properties. The following design cues were developed from the themes respectively; materials and design with high UVR protection; ability to customise with own headwear; ventilation to reduce steaming up; acoustic functionality to enable hearing and speech; foldable, portable, and easy to put on and take off.

**Conclusions:**

It is important to understand people’s experiences of using medical devices to improve their safety, efficiency and user satisfaction. The user experience themes and design cues, informed the iterative development of low fidelity visor prototypes as part of a codesign process. These design cues and responses to the prototypes are guiding commercial manufacturing and regulatory approval. The visor can then be prescribed to patients, providing an equitable service of care.

## Background

**Fig. 1:**
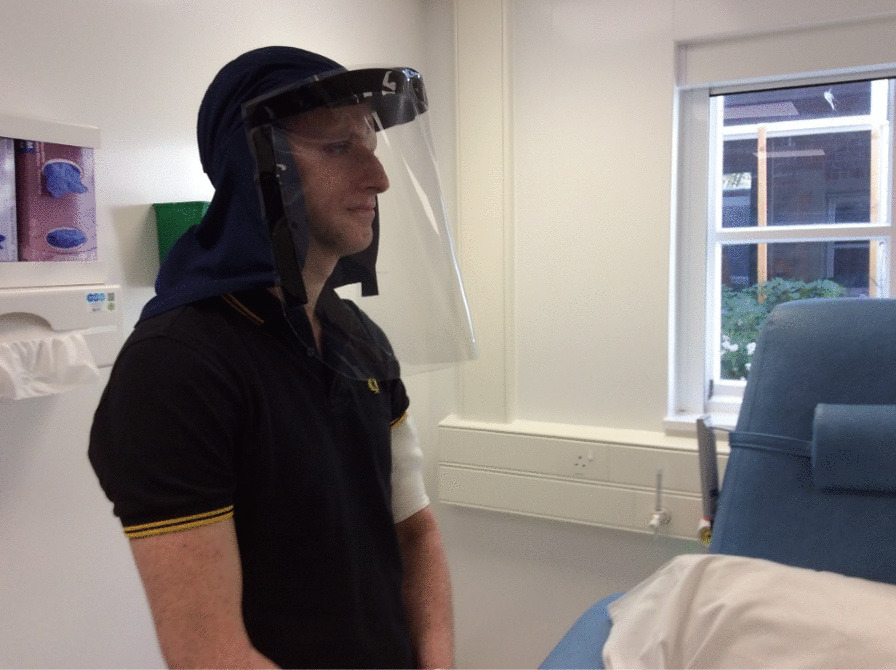
Current custom-made XP protective visor

Xeroderma Pigmentosum (XP) is a rare, genetic, incurable condition characterised by extreme skin sensitivity to daylight [1]. Incidences have been identified in all continents and racial groups at approximately 1, 2.3, 17.5 and 45 people per million live births in the United States, Western Europe, Middle East, and Japan respectively [2, 3]. Affected individuals have a 10,000-fold increased risk of developing skin cancer before their 20th birthday compared with the general population [4]. Some people with XP are also susceptible to severe sunburn with blistering and hyper or hypo pigmentation of exposed skin [1, 5]. As a result, management of the condition requires strict lifelong photoprotection [6, 7]**.** This encompasses minimising time spent outdoors during day light, repeat applications of factor 50+ sunscreen and wearing protective clothing that covers the entire skin surface, including Ultraviolet Radiation (UVR) protective glasses and full-face visors [1] (Fig. 1).

Currently, visors used by people with XP in the United Kingdom (UK) are homemade and comprise a UVR‐blocking plastic shield to cover the face. This is attached to a hat also made of UVR-blocking material to protect both the head and neck area [7] (Fig. 1). There is also a visor which provides acoustic and ventilation functions in an active battery-operated system [8], but this is not widely used in the UK (Personal communication).^1^ Despite the need for absolute protection from UVR, a third of patients’ face-photoprotection is sub-optimal [9, 10]. A minority of adults wear a visor (32.4%), whereas a large proportion of children and those under parental care do so on sunny days (85.9%) [11]. Even where there is a high level of adherence to photo-protecting, wearing the visor is reluctantly undertaken. Challenges include feeling uncomfortable alongside unwelcomed comments and negative feelings about being ‘different’ [12, 13].

We were tasked with codesigning a novel visor prototype to improve upon the current design and efficacy as part of a larger project to provide a commercially manufactured visor, regulated as a medical device that can be prescribed by clinicians. Understanding experiences of using medical devices, such as a photoprotection visor, is pivotal to ensuring they are safe, effective, and acceptable to patients [14–16]. User needs help to define the problem that requires a solution through design and collaborative working with other experts, a practice known as co-design [17, 18]. This paper presents the translation of user experiences and attitudes towards wearing a visor into design cues and prototype features as part of the codesign process. The process was underpinned by a participatory approach involving patients, carers, clinicians, researchers, medical physicists, engineers, designers, and industry partners. Full details of the engineering processes and bench testing will be published separately.

## Methods

Interviews were conducted at a Specialist XP clinic. The study sample was purposely recruited from the (approximately) 100 patients, registered with the service, and their carers. Invitations to participate in the interviews were also sent out by two charities supporting individuals and families affected by XP (The National XP Support Group and Action for XP). It was important to understand people’s experiences of wearing a visor and how these may have influenced their photoprotection choices. In addition, parents play an important role in influencing their child’s behaviour and strategies to manage their condition. Therefore, all patients diagnosed with XP were invited to participate and their families, regardless of whether they wore a visor or not.

**Fig. 2:**
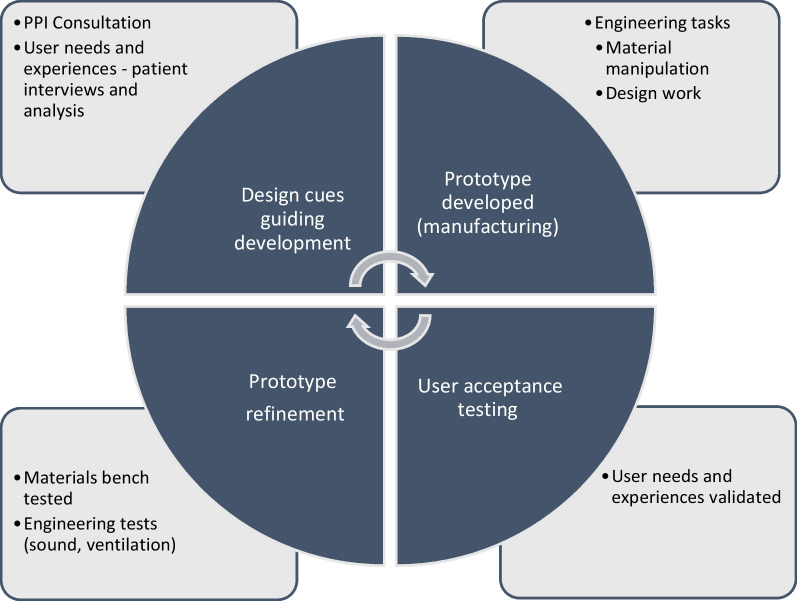
The codesign process—iterative development of prototypes through consultation, interviews, engineering, and bench testing

Based on patient and public involvement (PPI) consultations before the study began, an initial prototype was developed. Patients and their families were shown this first prototype followed by consecutive visor prototypes in the UVR-protected environment of the XP clinic after or between their scheduled clinic appointment(s). Following a topic guide, developed from the PPI work, participants were asked about their past and current and experiences of wearing a visor (or not); if they experience any problems with their current visor; to provide comments on the prototypes and suggestions to improve prototype materials and designs. Data collection took place in parallel with the analysis (as described below and illustrated in Fig. 2).

Interviews were audio-recorded and conducted by SS, TG, YSC and PG between December 2018 and November 2019. Each interview was transcribed, and anonymised by SS with edits made by TG, YSC, and PG. The researchers had between 5-and 20-years’ experience of conducting qualitative interviews with patients and families. Some of the participants knew about the study and had met the research team through the earlier consultations that took place whilst preparing the funding application. Facetime and Skype interviews were also conducted with families who wanted to comment on the prototypes as they progressed between clinic appointments. Interviews lasted between 20 and 90 min. One family viewed the prototypes whilst being visited by the XP Clinical Nurse Specialist at home. Academic engineers (SH and TM) were present in three of the patient interviews. An XP Clinical Nurse Specialist was present at four of the interviews.

### Translating users’ requirements into design cues through thematic analysis

After each interview, transcripts were read through and coded by (SS and TG). We undertook an inductive thematic analysis [19] guided by the following concepts of medical device development and evaluation: safety, efficiency and satisfaction [20]. We followed an interpretivist approach [21], to ensure the findings were grounded in the experiences of people living with XP. Using NVIVO 11 software, similar codes were then grouped to form themes categorising patient and carer's experiences. Each theme was scrutinised to ensure the data were mutually exclusive and consistent within each one. Data collection and analysis continued until no new themes emerged. Themes were interpreted into design cues; desirable attributes of a visor that would counteract the negative user experiences and meet the requirements described by patients and carers during the interviews [22, 23]. The aim of the analysis was to guide the development of the visor, not to generate theory. Design cues and their corresponding themes were presented back to patients during the interviews. Participants endorsed the themes and design cues providing respondent validation of the analysis [24]. The academic engineers (SH and TM) used the design cues to understand patient’s requirements and develop the functional and aesthetic components of the prototype designs during team meetings [15, 25]. Prototypes were shown to patients and their families and iteratively refined by the engineering team [26, 27] (Fig. 2).

## Results

**Fig. 3:**
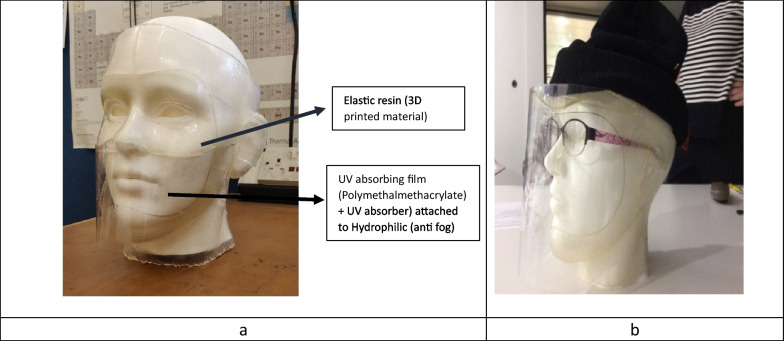
Images of prototypes. All prototype visors were designed and manufactured at Cardiff University. **a** Prototype 2 ‘On the face’—3D printed parts sealed together with hydrophilic film over the mouth. **b** Prototype 3 ‘Off the face’—rim added to increase space away from face

**Fig. 4:**
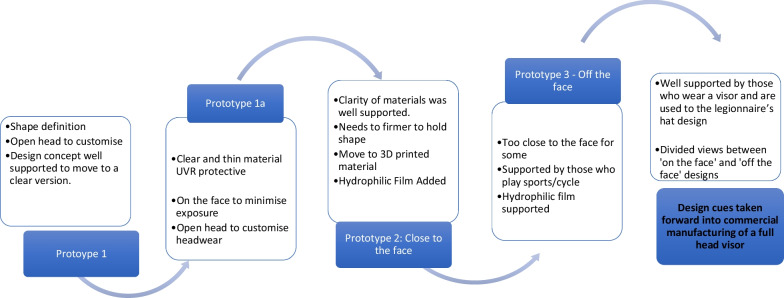
Iterative development of prototypes with testing results and refinements

Twenty-four interviews were conducted with patients and their families (Table 1). Although we offered interviews to all the 100 patients attending the clinic, there were a range of reasons that some were unable to participate. Some patients had to attend other appointments and some, who did not wear a visor, declined our invitation to review the prototypes. Thematic analysis resulted in the following five themes: Being safe from UVR exposure, self-consciousness; temperature effects; acoustic difficulties; and material properties. The following design cues were developed from the themes respectively; materials and design with the highest possible UVR protection; ability to customise with own headwear; ventilation to reduce steaming up; acoustic functionality to enable hearing and speech; foldable, portable, and easy to put on and take off (Table 2). Guided by these design cues, two prototypes were proposed by the academic engineers: one ‘on the face’ and one ‘off the face’ (Figs. 3a, b and 4).

**Table 1:** Demographic details of participants

Participants	Patients aged 16 and above (Adults)	Patients aged 11–15 (Young people)	Patients aged 5–10 (Children)	Parent of patient aged 16 and above	Parent of patient aged 5–15 (carer)	Parent of Adult patient who needs complete care
Number of participants	16	3	5	9	11	1
Gender	13 Males 3 Females	1 Males 2 Females	3 Males 2 Females	3 Males 6 Females	5 Males 6 Females	Female
Genotype-Phenotype Code	XPA-1, 2 XPA-1 XPA-3 XPC-2 XPC-2 XPC-2 XPC-2 XPC-3 XPC-3 XPC-3 XPC-3 XPV-3 XPV-2 XPD-1,2 XPE-2 XPF-1	XPC-1 XPC-3 XPC-3	XPA-3 XPC-3 XPC-3 XPC-3 XPD-3			
Number of participants who wear a visor	Yes 8 No 8	Yes 3	Yes 5			

Ethnicity: Twelve (patient) participants were White Caucasian and 12 were either Asian, Arab, or Other

Phenotype Code: 1- Diagnosed with a neurological condition, 2- Diagnosed with skin cancers, 3- Neither neurological condition or skin cancers diagnosed

**Table 2:** Translation of user needs to prototype visor

User experience theme	Codes:	Illustrative quote (Q)	Design cue	Prototype features
Being safe from UVR exposure	Lack of confidence in home-made visors	*Her existing visor is a grey baseball cap made by her aunt with an attached although not securely, by circular velcro stick-ons, meaning there are holes in the design that may allow for UV exposure…*(Mother of patient aged 11–15)	Materials and design with the highest possible UVR protection	Prototype materials developed comprising clear silicone with added UVR absorbers; elastic resin; rapid 3D printing of materials to manufacture prototypes
				On the face design
	Seals and gapping			Close to the shape of the face to reduce gapping around the face and neck
	Thought they were protected but then got sunburnt e.g. UVR reflected from water or snow			Off the face as alternative design
	Not going outside to avoid UVR exposure			Wrap around design secured with strong magnets at the back
Self-conscious when wearing current visor	Being stared at; bullied	*So yes, you want it to be safe, you want it to be practical. But it’s also, what I feel is the biggest barrier, it’s whether or not you can deal with how much attention it draws* (Adult patient)	Ability to customise with own headwear	Open at the top to give the choice of personalising with own headwear (securely fixing using provided magnet strips)
				Clear material so the face is easily seen
	Using alternatives to the visor to photoprotect	*I don’t want to attract the entire [name of supermarket] looking at me. I don’t want to attract a lot of attention…a normal boy who is wearing something really weird… mmm…that feels weird* (Patient aged 5–10)	Aesthetically acceptable	Patients have the option to wear their own glasses or sunglasses
Temperature effects	Heat management	*It can get hot in the summer and it just mists up all the time and I cannot see anything* (Patient aged 11–15)	Ventilation system to enable breathing/temperature control/steaming up	Engineered and tested a passive acoustic/ventilation system under laboratory conditions
	Difficult to breathe			
	Visor steaming up			
	Drinking outdoors is not feasible without UVR exposure			
	Not going outside to avoid heat	*No matter what the weather she is always sweating… she gets very, very hot. Even in winter, she goes to school a little bit further away from home and I bring her back on transport either by cab or whatever, depends on the weather but even five minutes with this on she ends up with rashes from the heat* (Father of patient aged 5–11)	Clear materials that do not steam up	Hydrophilic film around the mouth area to reduce steaming up
Acoustic difficulties with current visor (speech and hearing)	Difficulty being heard; hearing others	*The sound rebounds back in the visor back to me. I have to lift it slowly up to my nose and then talk* (Adult patient)	Acoustic system to enable hearing and clear speech	The ventilation ports with three overlapping flaps allowed sufficient sound to penetrate through
	Loss of confidence			
Material properties	Difficult to fold	*… with the current mask that we’ve got, if I need to put in my bag, it’s quite stiff. If I bend it too much, then it gets holes in it* (Adult patient)	Foldable and portable	Material is thin and light, does not leave creases when folded
	Difficult to transport			
	Difficult to use	*… they (patient’s current visors) don’t fold. They fold somewhat easily, more easily if they get hot, they just lose their shape* (Adult patient)	Easy to take on and off	

The engineering solutions are outlined to illustrate their relation to the user needs analysis. Full details of the manufacturing processes, bench testing of materials and engineering functions will be published in a separate paper

### Being safe from UVR exposure: the need for materials and design with high UVR protection

Photoprotection was described as a way of life for people living with XP. All 5–15-year-olds and half of adult participants wore visors (Table 1). Of the seven adults who had experienced skin cancers, three wore a visor. Half of the adults with a neurological condition, wore a visor and of the six adults who experienced neither skin cancers or neurological conditions, four wore a visor. Adults who did not wear a visor wore other types of photoprotective headwear (e.g. hat/hoodie/scarves). Participants who wore a visor would also combine this with other forms of photoprotection including neck buffs and sunglasses. Some participants had UVR protective film on windows at their home, work or school and in their cars. Other participants would avoid going out during the day, especially when it was sunny, waiting until the evening to go outdoors. Most participants wore sunscreen either alone or in addition to other photoprotective clothing, glasses and visors. This was often described as an onerous task given the need to reapply sunscreen several times during the day:

‘Yeah I go out. But I’ve got to wear my sun cream obviously. But even just like, somebody could just say I’m going to nip to the shops for some milk, I can’t do that. I’ve got to apply cream before I go anywhere. It takes ages. It takes ages to get ready. You’ve got to wait for it to dry. It’s terrible'.

(Adult patient).

All parents described custom making visors following the legionnaires hat design (See Fig. 1). Adults also made their own visors with help from their family members and learned techniques from childhood. One parent who runs one of the XP patient charities noted they were often tasked with attaching the UVR-block plastic to the hats and sending out the visors ready-made to families. As well as ensuring protection for the front of the face, it was important to also cover the back of the neck:

‘I bought the material online and it got patched together by the seamstress. The hat was bought without this insert in it, the material was bought separately because it is important to put extra fabric to get the neck coverage’.

(Adult patient).

Parents went to great lengths to ensure their child was protected, trying a range of different devices and materials but did not know the level of protection they provided. There were instances when parents thought their child was fully protected but then realised, they had been exposed either through a gap in their visor or light reaching an area not protected by the visor (e.g. under the neck). This led to vigilant behaviours to confirm UVR did not enter custom-made visors:

Father: They come ready made [from the patient charity] with the round Velcro, it’s similar. I cut my own and glue the strips.

Mother: Because when we made the first one, it was like holes in there.

Father: And then I checked them with the dosimeter and it does come through. Even that, I am sure that’s why I made them with roll-neck…

(Parents of a patient aged 5–10).

### Material properties: the importance of durability and portability

Visors were manipulated frequently as they were put on, taken off and carried around throughout the day. Given people would alternate between being inside and outdoors, it was important to be able to put their visor on and take it off easily. Some participants reported that their current visor material was very stiff, would lose shape and get easily damaged. Visors were replaced about twice a year. Other participants noted that their current visor material was rigid and heavy rendering it difficult to fold and carry around. About half of the participants (majority being parents) said having a thin and flexible material was important:

‘Yes, I like this [candidate material]. There is more flexibility there which is good. Whereas, what they are wearing at the moment is so rigid. Like at school and things, she’s folding her hat up now and putting it in her bag so that’s she carrying it around all day, folded up in her bag…’

(Mother of patient aged 11–15). 

### Engineering solutions: manufacture and durability of UVR protective materials

Guided by the need for high UVR protection and durability the academic engineers developed a range of novel clear silicone materials with added UVR absorbers. The materials were tested by medical physics experts and were found to be over 99.9% UVR protective and able to withstand extreme weathering (personal communication).^2^ However, when the engineers attempted to manufacture the material into the shape of the visor it was too thin to support the visor shape. The engineers then changed the material to a commercially available thicker material that could be manipulated to hold the prototype shape through 3D printing. The 3D printer was not large enough to print a visor in one piece, so the component parts needed to be sealed together (Fig. 3a). The seals made the visor look more obvious for some participants and others did not like the material being so close to the skin, especially when they use sun cream:

‘It feels like wearing a mask…it is very strange. I don’t look like myself in that. That is quite distorting to your face and so if it is pulled away from the face slightly...’

(Mother of patient aged 5-11)

‘I wear a lot of sun cream and moisturiser, so I suppose I’d be worried about it getting too up close to my skin, and it making a sticky mess, and it might not look great’.

(Adult male patient).

In response to these views, a rim was added to the forehead area to allow more space between the face and the visor (Fig. 3b). Although Prototype 3 ‘on the face’ concept was designed to reduce gapping around the face and neck, and featured wrap around glasses, some participants were concerned about UVR exposure between the areas that were sealed together. With the proviso that there would be a UVR tight seal between the component parts, some considered Prototype 3 to be useful for playing sports and preferred the close fit to the face:

‘Just looking at it, I’d say it looks pretty amazing. I would always just always say, thinner the better, so just it’s not too out there. It would just make him more comfortable, rather than people staring. Because with the current visor, it is an issue, it stands out a fair bit. So I am thinking with this, which would hopefully be quite snug, so with a cap you would barely see it, but it also has to be comfortable for him, that would be the priority’.

(Father of patient aged 5-11) 

### Self-conscious when wearing visor: the ability to customise with own headwear

Educating others on the need to wear a visor was commonplace. Some participants said that people at their school, college or workplace understood their need to wear a visor however they still felt different to others. Wanting to look normal and fit in was considered important. Adult participants who eschewed wearing a visor explained that they did so because it was too conspicuous:

‘You know what with wearing one of them, people know that you are different, and I don’t want that. I want to be like everybody else. Have a normal life. I know I can’t but try and have a normal life. But if you wore things like that, no way’.

(Adult patient).

All the adults, young people and children, who wore a visor, said they received negative attention when wearing their visor. Most reported that other people stared at them, and some said they had been bullied. Adult and parent participants explained that children were much more likely to wear a visor when under the influence of their parent but once they entered teenage years, they became more aware of their appearance, and some chose not to wear a visor:

‘Because she was a young female, it was ugly looking, and people would stare at her and call her names. Children are cruel when they are growing up. So, with the visor being uncomfortable for her to wear, that is why when she got to adulthood, she made the decision not to wear it anymore’.

(Parent of adult patient).

Adults negotiated UVR exposure and appearance in a range of different ways. For some adults, wearing a visor was weather dependent and they would only do so if it was ‘really hot and sunny’. Other adults preferred to wear alternative forms of face protection to avoid being stared at (hoodies, glasses, scarves). However, these forms of protection could also attract negative attention and look suspicious:

‘So then, when I was a child I used to wear it religiously and then there was a period of time, maybe when I was 17, 18 or 19 where I really did not like the look of it so I use to try and come up with all different solutions myself and what I just settled on was wearing a scarf that covers up to my eyes with sunglasses and a very deep hood that would come over my eyes. So, it may have been a little bit inferior protection but looking back, you still got a lot of stares because it looked a little bit thuggish’.

(Adult patient).

### Engineering and design solutions: customising headwear on the visor

One way of reducing conspicuousness was to customise the visor with headwear and glasses chosen by the patient themselves. Most participants supported this design with the proviso, the seal was intact between their headwear and the visor itself to ensure there was no exposure. This was particularly important for parents who noted that visors would easily come off when children were playing:

‘They had the bulk of the mask, the lower level, separate from the top and I was very concerned that if the patient tries to put this on, then there has to be 100% mechanical connection between the top and the bottom so that no UV can ever get through under any circumstances’.

(Father of adult patient).

Most of the participants lived active lives. Some were keen cyclists and others enjoyed outdoor sports such as fishing. They noted that customising the visor gave them the option to choose different hats according to the season and activities they were taking part in:

‘I think that the component would… it would be good if you could attach it to your cap. Because some people do not like the way it looks, so with the visor because I didn’t like it it’s look, but it could become more fashion, you could just have the head part detachable, so people could have the option to wear it with this and have the option to wear a normal baseball cap and put it under there. At least it is not coming through, you still have the protection’.

(Adult patient).

Being able to choose different coloured material covering the neck and add attachments such as stickers was also an important option for young people and children who wanted to personalise their visors.

### Temperature effects: the need for a ventilation system to enable breathing and reduce steaming up

All participants (adults, children, young people and parents) said that current visors steam up and feel very hot when wearing them. Some also said it was difficult to breathe when wearing a visor:

‘I find it hard to breathe you can’t breathe through your nose…the number of times when I am walking, I struggle to breathe within the first three minutes I think I am going to faint and be so hot’.

(Patient aged 5–10). 

Feeling claustrophobic was a common problem. Some said they avoided going outside during the day because they felt too hot under a visor, especially during the summer. Breathing directly onto material that is close to the face also led to condensation and steaming up, and difficulty seeing through the material:

‘Yes, there are lot of issues…. when you’re wearing it. For the first ten minutes everything is fine, but you feel like you are suffocated, and it gets all steamy inside. The worse thing about it; you have to keep taking it off in broad daylight and clean it and while you are cleaning it, it lets UV come onto you, so it’ll still affect the skin somehow. You have to keep wiping and putting back on’.

(Adult patient).

Staying hydrated whilst wearing a visor was important given the heat issue especially during warmer months. About half of our participants commented that they had difficulty drinking when outside given the need to lift the visor which would lead to UVR exposure:

‘We have never really thought about eating because with the hat that she’s wearing now, it’s just second nature that we just go in somewhere and we just eat and drink. And mainly just for that reason, she cannot do it otherwise you are tipping the hat and the UV is getting in; we just cannot even go there’.

(Mother of patient aged 11–15).

### Acoustic difficulties with current visor: the ability to hear and be heard by others

In addition to looking and feeling different, wearing a visor could also impact on communication. Half of the participants noted that hearing what others were saying was problematic when wearing a current visor. Parents, young people and adults who wore visors said they experienced an echo or muffled sound when they spoke. Some parents also said that when their child was speaking, others found it difficult to hear what they were saying.

‘I cannot hear her. Her teachers find it quite hard to hear her with the mask on and a couple of times I have been on the trips, I’ve gone with them and on the way back the teacher can’t hear what she is saying. But even her, it’s the same both ways, I can tell her something and she won’t hear. I have to go right close; it sounds a bit, it’s got an echo. It sounds a bit like Darth Vader’.

(Father of a patient aged 5–11).

Problems with hearing others and being heard impacted on the ability to socialise and interact with others. Young people and adults said that they could only hear when someone was speaking close to them. One participant described how, when wearing a visor, her daughter was unable to take part in conversations:

‘I can’t hear anything anybody says to me’.

(Patient aged 11–15)

‘Friends are right next to her and talking to her one to one, she can’t hear what’s going on so she just has a tendency to shrink into the background and just let everyone else get on with it really’.

(Mother of a patient aged 11–15).

### Engineering solutions: development of ventilation system to enable breathing and reduce steaming up

To counteract overheating, the academic engineers developed and tested a passive ventilation system under laboratory conditions. The aim was to establish a steady air flow into and out of the visor while simultaneously absorbing and blocking UVR through light traps. Laboratory tests also showed that no UVR leaked through. Finally, the air flow through the ventilation ports was found to improve when a breeze was simulated (e.g. running with the visor on). Participants appreciated the difficulties of balancing the need for air flow without allowing any UVR in or exposure:

‘The idea of the flaps [light traps] are good and the engineers still need to find a way to make sure that the ears are still protected but it’s like what I said before, they need to stop UV getting in but let the ventilation come in’.

(Adult patient).

To address the issue of misting and steaming up, the mouth component was made of an UVR absorbing film attached to a commercially available hydrophilic (anti fog) film (see Fig. 3a, b). The film did not steam up when blown on or used to speak through. Participants were impressed by the anti-fog, but concerns were expressed that UVR could enter through the small air bubbles which were visible, having been trapped between two layers of the material during manufacturing:

‘Yes the heat, and the steaming. So, this with the new technology would be fantastic, because it won’t steam up, and the new system and more ventilation, then he will obviously be able to stay out longer…’

(Father of patient aged 5–11).

Some participants reported hearing to be less of an issue compared to other functions such as over heating or steaming up. However, given the importance of hearing on communication an ear component with over lapping openings was incorporated into the visor prototype within the ventilation ports as described above. Tests indicated that sufficient sound was able to penetrate through. Being able to take part in conversations was noted as important by one young person who felt the ear openings were an important feature of the visor design.

## Discussion

The aim of this study was to co-design novel visor prototypes by understanding people’s experiences and attitudes towards wearing an XP visor. Design cues were developed from the following five themes: safety from UVR exposure, self-consciousness; temperature effects; acoustic difficulties; and material properties. It was important that prototypes were made with materials with the highest possible UVR protection and had a UVR tight seal between components. Materials needed to be easy to carry and manipulate into the visor shape. Being able to customise the visor with own headwear was also desirable. Overheating and hearing difficulties were addressed through ventilation and acoustic functions designed into the prototypes.

### Appearance of the visor

Visors are arguably the most conspicuous type of photoprotective wear given they are worn on the face and cover the head and neck. The finding that people received negative attention when wearing a visor and try to minimise looking different has been reported in other qualitative studies of people with XP [12, 13, 28]. Not wanting to feel different has also been reported by people with other chronic conditions, who use visible therapeutic devices such as bandages [29, 30]. Aesthetics is an important part of user satisfaction especially for devices that are carried around and regularly used such as handheld devices for airway clearance, used by people with Cystic Fibrosis and Chronic Obstructive Pulmonary Disease [31]. In our study, we acknowledge that some people felt strongly against wearing a visor largely because of the way it looks. For these people, this project may not change their decision, and indeed that was not our intention. Our brief was to co-design a visor to counteract the limitations of existing head wear. We understood we could never make it look as if someone is not wearing a visor at all. However, having clear materials that give prominence to the face and headwear of someone’s choice to attach to the visor was one proposal to *normalise* the look.

Interestingly, some people who did not wear a visor wanted to participate in the research. They may have wanted to see what was being designed and be involved in improving the visor design through sharing their experiences. Our study was undertaken before the COVID-19 pandemic. The stigma attached to wearing a visor may have been lessened during this time as wearing a face covering has become more accepted. Future research can investigate the impact of the pandemic on the experiences and attitudes of visor wearing among patients with XP and their carers.

### Adapting behaviours to manage risk and participation in social activities

Alternative forms of headwear served to minimise stigma but also brought increased risk of exposure. This illustrates how participants negotiated risks between their appearance and exposure to UVR. Some were risk averse and were more willing to deal with the (potentially) negative attention associated with wearing a visor to ensure they were protected from UVR exposure. Previous studies with people with XP have also found that people choose to override the negative feelings of stigma associated with photoprotection and wear a visor to protect themselves [12, 13]. Other people in our study were willing to take the risk of being exposed in return for feeling more comfortable in how they looked. They chose not to wear a visor but other forms of photoprotection (hat, sunglasses, hoodie) in addition to avoiding outdoors at different times of the day.

Although visors can facilitate outdoor activities, individuals had to negotiate several threats to participation. Wearing a visor limited the quality of social interactions and the length of time someone could be outdoors due to poor temperature control and ventilation. Individuals had to regulate their exposure to UVR by avoiding going outside during the day or moving inside to drink or have breaks. This is supported by a recent observational study that reported adults with XP achieved photoprotection to the face by reducing the time they spend outdoors and not through wearing photoprotective items [32]. Adapting to the constraints of living with a chronic condition is an integral part of the coping strategies developed by families [33, 34]. Having to adjust family life and social participation, particularly when a condition cannot be seen by others, can create a perceived difference between people with rare conditions and those without [35]. Designing prototypes which improve ventilation and acoustics may enable families and adults to better negotiate the limitations of their social lives and activities [36].

### Negotiating user needs and managing expectations in the codesign process

Key mechanisms underpinning the codesign process involve recognising users’ needs through dialogue and enacting change through cooperation and accountability [37, 38]. However, there are several factors that influence how this knowledge is translated into prototypes [39]. Firstly, some combinations of the design cues were contradictory. For example, having a material that was thin enough to enable flexibility with a high UVR protection and simultaneously hold the visor shape was challenging. In addition, preventing UVR from penetrating the visor whilst allowing air and sound to circulate was difficult. The codesign process therefore requires consideration of how patients’ needs map onto what is technologically feasible, and clinically significant.

Secondly, exploring the way that people relate to medical devices can not only reveal relationships between people with the condition and the device itself but also between different members of the codesign team [40]. The patients and carers priority were for a safe visor that is visually acceptable, does not steam up or interfere with communication. For the XP clinical nurse specialists, who inspired and collaborated in the study, the priority is for a prescribe-able standardised UVR head protecting visor that is acceptable to patients to enhance safety and provide an equitable service of care. There is a slight disparity as well as overlap with these two priorities illustrating the intersection of responsibilities, experience and motivations of different stakeholders engaged in the codesign process [41]. If the visor were customised with own choice of hat, the level of photoprotection would not be guaranteed. This would threaten the potential for the visor to be prescribed for UVR protection given there would be an unknown variable (own hat). To exclude the impact of variation through personal choice of hat, the clinicians requested a full head visor to ensure complete photoprotection.

Thirdly, when patients are involved in designing a new product they also want to know when they might be able to use the devices themselves. The strategy we adopt is to be open and transparent about the delivery of the outputs. For example, the patient information sheets stated that the devices would not be available in the lifetime of the funded study and required further commercial development. We continued clear communication when there were setbacks, for example interruptions to prototyping during to COVID-19, the re-planning to mitigate these and not giving up on the goal of translating their needs into commercially available devices [42].

### Strengths and limitations

The XP population is small and spread across the UK. Most patients attended multiple clinical appointments during their hospital visit so the least burdensome way for them to participate in our study was in between or after their appointments were completed. Therefore, it was not possible to employ age-appropriate methods for interviewing children, such as drawing and game playing which require more time. In addition, it may have been more appropriate to interview children without their parents, so children were able to be more open about their views. However, because parents are pivotal to their child’s photoprotection regime, we were interested in their experiences as well as those of the children. We found that instead of impeding responses, parents prompted their children to discuss their experiences and views of the prototypes [43].

This study focused on exploring experiences to guide the development a visor for people with a rare genetic skin condition. The findings are therefore not necessarily applicable beyond people with XP or wearing a visor for photoprotection. However, this study provides an example of how patients and carers experiences, unmet needs and preferences are being translated into designing a novel photoprotection visor whilst also considering the clinical imperatives for total protection from UVR. This contributes to the growing evidence-base of how to codesign medical devices and manage critical differences in expectations and preferences [44–46]. Even though the population of people with XP is relatively small compared to other more common conditions, we were able to recruit approximately a quarter of the UK population (24/100) and their parents to provide their views and experiences of wearing a visor and the codesigned prototypes.

### Conclusions

Understanding how devices are used in the real world and not only in scenarios re-enacted by designers can improve their safety, efficiency, and satisfaction [47, 48]. This study contributes to our understanding of user requirements for a novel photoprotection visor and of the role that co-design plays in the development of effective devices. Hearing about people’s experiences of wearing a visor and photoprotective clothing illustrate how they negotiated exposure to UVR. These experiences were translated into design cues to guide the development of prototype visors. The design cues and learning accrued in this study are being translated into functional prototypes for commercial manufacturing by a UK Catapult, The Manufacturing Centre. Catapults are not for profit organisations that link universities with industry to commercialise research outputs to ensure patients get the products they helped to co-design. The next steps are to identify a commercial manufacturer and distributor, to conduct patient usability tests and a cost analysis of the commercial prototypes which meet medical device regulatory approvals. This will lead towards meeting the goal of a commercially manufactured visor for people with XP that can be prescribed through UK and International XP support groups and health services, providing an equitable service of care.

## Data Availability

Due to the rarity of Xeroderma Pigmentosum, the UK population is small (n = 100). Therefore, it is difficult to sufficiently anonymise interview transcripts to go on an open access data repository. Any reasonable requests for necessary information required by a reader or reviewer will be granted by the authors.

## Footnotes

1 Clinical Nurse Specialists, Sally Turner and Tanya Henshaw from the National Xeroderma Pigmentosum Service in Guy’s and St Thomas’s NHS Foundation Trust, London.

2 Aneju G, Fedele F, Freeman P, Hashimdeen S, Meydan T, Grocott P: Assessment of a Newly Developed UVR Absorber Material Sample for Xeroderma Pigmentosum (XP) UVR Protection. IPEM Optical Radiation Update Conference, Oct 2020.
